# Chronic ischemic mitral regurgitation and papillary muscle infarction detected by late gadolinium-enhanced cardiac magnetic resonance imaging in patients with ST-segment elevation myocardial infarction

**DOI:** 10.1007/s00392-016-1006-9

**Published:** 2016-06-08

**Authors:** Wobbe Bouma, Hendrik M. Willemsen, Chris P. H. Lexis, Niek H. Prakken, Erik Lipsic, Dirk J. van Veldhuisen, Massimo A. Mariani, Pim van der Harst, Iwan C. C. van der Horst

**Affiliations:** 1 Department of Cardiothoracic Surgery, University Medical Center Groningen, University of Groningen, P.O. Box 30001, 9700 RB Groningen, The Netherlands; 2 Department of Cardiology, University Medical Center Groningen, University of Groningen, Groningen, The Netherlands; 3 Department of Radiology, University Medical Center Groningen, University of Groningen, Groningen, The Netherlands; 4 Department of Critical Care, University Medical Center Groningen, University of Groningen, Groningen, The Netherlands

**Keywords:** Myocardial infarction, Papillary muscle infarction, Mitral regurgitation, Echocardiography, Magnetic resonance imaging

## Abstract

**Background:**

Both papillary muscle infarction (PMI) and chronic ischemic mitral regurgitation (CIMR) are associated with reduced survival after myocardial infarction. The influence of PMI on CIMR and factors influencing both entities are incompletely understood.

**Objectives:**

We sought to determine the influence of PMI on CIMR after primary percutaneous coronary intervention (PCI) for ST-segment elevation myocardial infarction (STEMI) and to define independent predictors of PMI and CIMR.

**Methods:**

Between January 2011 and May 2013, 263 patients (mean age 57.8 ± 11.5 years) underwent late gadolinium-enhanced cardiac magnetic resonance imaging and transthoracic echocardiography 4 months after PCI for STEMI. Infarct size, PMI, and mitral valve and left ventricular geometric and functional parameters were assessed. Univariate and multivariate analyses were performed to identify predictors of PMI and CIMR (≥grade 2+).

**Results:**

PMI was present in 61 patients (23 %) and CIMR was present in 86 patients (33 %). In patients with PMI, 52 % had CIMR, and in patients without PMI, 27 % had CIMR (*P* < 0.001). In multivariate analyses, infarct size [odds ratio (OR) 1.09 (95 % confidence interval 1.04–1.13), *P* < 0.001], inferior MI [OR 4.64 (1.04–20.62), *P* = 0.044], and circumflex infarct-related artery [OR 8.21 (3.80–17.74), *P* < 0.001] were independent predictors of PMI. Age [OR 1.08 (1.04–1.11), *P* < 0.001], infarct size [OR 1.09 (1.03-1.16), *P* = 0.003], tethering height [OR 19.30 (3.28–113.61), *P* = 0.001], and interpapillary muscle distance [OR 3.32 (1.31–8.42), *P* = 0.011] were independent predictors of CIMR.

**Conclusions:**

The risk of PMI is mainly associated with inferior infarction and infarction in the circumflex coronary artery. Although the prevalence of CIMR is almost doubled in the presence of PMI, PMI is not an independent predictor of CIMR. Tethering height and interpapillary muscle distance are the strongest independent predictors of CIMR.

## Introduction

Both papillary muscle infarction (PMI) and chronic ischemic mitral regurgitation (CIMR) are associated with reduced survival after myocardial infarction (MI) [1–3].

Ischemic mitral regurgitation (IMR) is a common complication of MI with an estimated incidence of 20–50 % [4–8]. IMR is frequent early after MI, but it is often mild and may disappear completely [4–8]. When IMR develops, persists or increases over the course of several weeks after MI, it becomes chronic [4–8]. Several studies showed that (even mild) CIMR after MI increases the risk of congestive heart failure and death in a graded fashion according to mitral regurgitation (MR) severity (independent of left ventricular (LV) function) [1, 2]. The exact mechanism for the development of IMR after MI remains a subject of debate [9]. IMR may develop acutely after post-MI papillary muscle rupture, or more gradually with scar formation, LV remodeling, papillary muscle (PM) displacement, and mitral valve tethering or tenting (i.e. CIMR) [9, 10].

PMI has a strong (negative) prognostic value after MI [3]. This may be related to the development of CIMR, but the precise role of PM involvement in the development of CIMR is still unclear. Factors influencing PMI are incompletely understood. Late gadolinium-enhancement (LGE) cardiac magnetic resonance imaging (MRI) enables the noninvasive detection of papillary muscle infarction (PMI) with high spatial resolution [3, 11]. Therefore, LGE cardiac MRI is the technique of choice for PMI assessment.

In this study, we sought to determine the influence of PMI detected by LGE cardiac MRI on CIMR after primary percutaneous coronary intervention (PCI) for ST-segment elevation myocardial infarction (STEMI) and to determine independent predictors of PMI and CIMR.

## Methods

### Study Design

This study was performed as a substudy of the glycometabolic intervention as adjunct to primary percutaneous intervention in ST elevation myocardial infarction (GIPS)-III trial (clinicaltrials.gov NTC01217307) [12–14]. The GIPS-III trial was a prospective, single center, double blind, randomized clinical trial that compared metformin 500 mg twice daily to placebo treatment in 380 non-diabetic patients requiring primary PCI for STEMI. The primary endpoint, left ventricular ejection fraction (LVEF) after 4 months, was similar between groups [13]. The final results of the GIPS-III trial have been reported previously [13]. In brief, patients aged ≥18 years presenting with a first STEMI and undergoing primary PCI with implantation of at least 1 stent with a diameter of at least 3 mm resulting in thrombolysis in myocardial infarction (TIMI) flow grade 2 or 3 post-PCI were included. Major exclusion criteria were known diabetes, the need for coronary artery bypass grafting, severe renal dysfunction, and contraindications for MRI. All patients provided written informed consent. The study protocol was in accordance with the Declaration of Helsinki and was approved by the local ethics committee (Groningen, the Netherlands) and national regulatory authorities.

### PMI substudy

Between January 2011 and May 2013, 380 patients were enrolled in the GIPS-III trial. A total of 275 patients underwent cardiac MRI and transthoracic echocardiography (TTE) 4 months after PCI. 263 patients had an evaluable cardiac MRI, and were eligible for the current substudy. None of these patients had a history of (organic) mitral valve disease.

Standard laboratory assessment including serum concentrations of creatinine phosphokinase (CK) was performed.

### Angiographic analysis

Coronary angiography and coronary intervention were performed using standard techniques. The choice and order of coronary intervention (i.e., thrombus aspiration, balloon angioplasty, or stenting) was left to the discretion of the operator. Perfusion was evaluated according to the TIMI criteria [15]. Myocardial blush grade was assessed for the infarct-related artery, and was defined as previously described [16]: 0, no myocardial blush; 1, minimal myocardial blush; 2, moderate myocardial blush; and 3, normal myocardial blush or contrast density. Persistent myocardial blush suggesting leakage of contrast medium into extravascular space was graded as 0.

### Cardiac MRI protocol

Cardiac MRI was performed 4 months post-PCI with a 3.0 Tesla clinical scanner (3 T Achieva, Philips, Best, the Netherlands) using a phased array cardiac receiver coil. Electrocardiogram-gated cine steady-state, free precision magnetic resonance images were acquired during repeated breath holds in the standard long-axis views (4-, 3-, and 2-chamber view) and contiguous short-axis slices of 1 cm covering the entire LV were used to assess global and regional ventricular function and to calculate LVEF. Using identical slice locations, late contrast-enhanced images were acquired 10 min after intravenous administration of a gadolinium-based contrast agent (Dotarem, Gorinchem, The Netherlands; 0.2 mmol/kg) with an inversion recovery, gradient echo pulse sequence to identify the location and extent of MI and PMI. The inversion time was set to null the signal of viable myocardium for every individual patient.

### Cardiac MRI analysis

Images were stored and sent to an independent cardiac MRI core laboratory (Image Analysis Center, VU University Medical Center, Amsterdam, The Netherlands) for assessment by fully blinded operators. Additional assessment of PMI and mitral valve geometry was performed using an open-source software package (OsiriX Imaging Software).

**Fig. 1:**
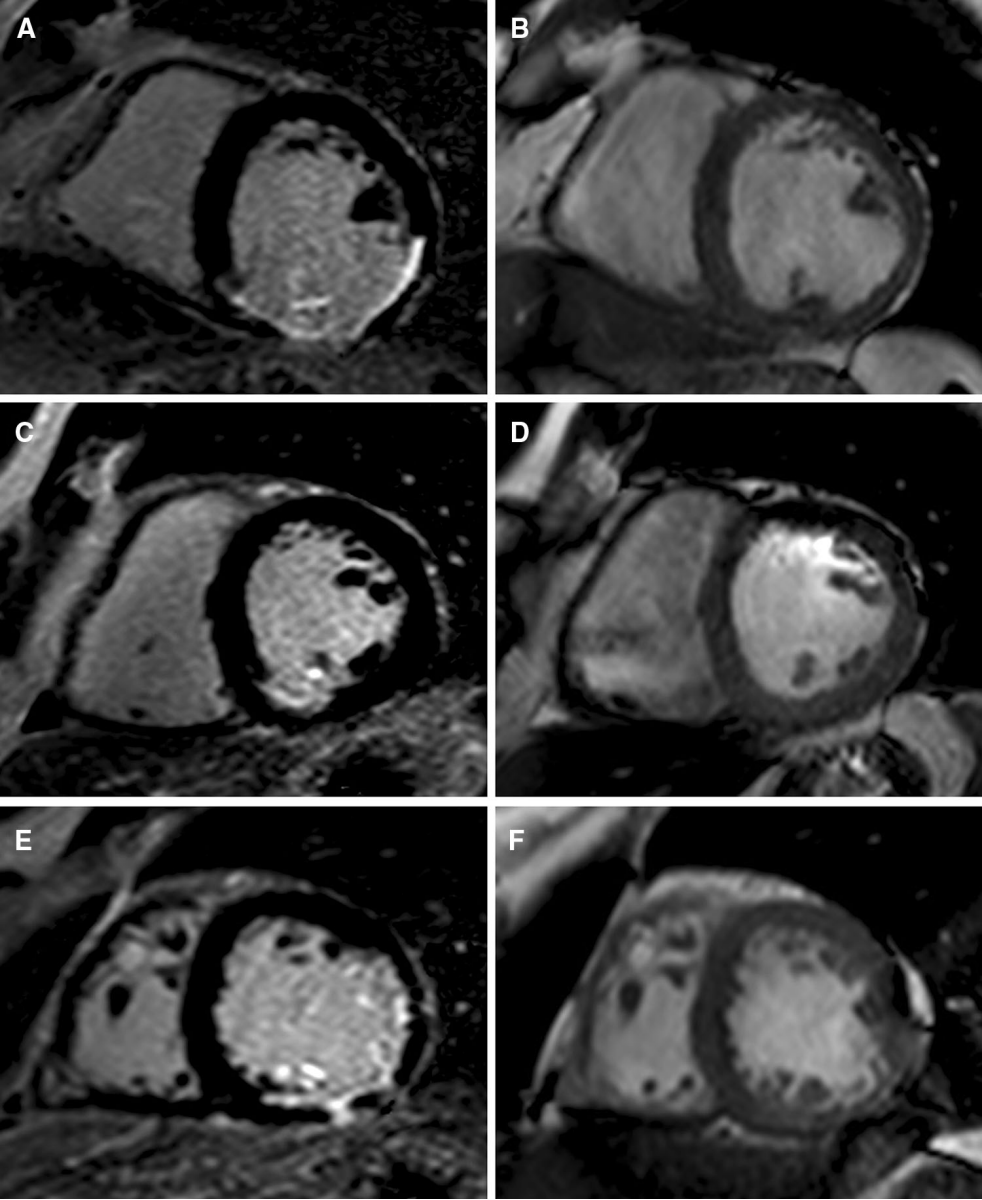
Assessment of PMI by LGE cardiac MRI. Patient with inferolateral STEMI with complete infarction of the posteromedian PM. **A** LGE image. **B** Cine image of the same location as a side-by-side reference for localizing the PM within the blood pool during interpretation of contrast-enhanced images. Patient with inferior STEMI and partial infarction of the posteromedian PM. **C** LGE image. **D** Cine image. Patient with anterolateral and inferior STEMI and combined partial infarction of the anterolateral PM and complete infarction of the posteromedian PM. **E** LGE image. **F** Cine image. *(AL)PM* (anterolateral) papillary muscle, *LGE* late gadolinium-enhancement, *MRI* magnetic resonance imaging, *PMI* papillary muscle infarction, *(PM)PM* posteromedian papillary muscle, *STEMI* ST elevation myocardial infarction

Summation of the volumes per slice of areas of hyperenhancement was outlined, allowing calculation of total infarct size (% LV myocardium). PMI was evaluated by LGE cardiac MRI images. Cine images of the same location were used as a side-by-side reference for localizing the PM within the blood pool during interpretation of contrast-enhanced images. PMI was considered present if any papillary hyperenhancement was present on LGE images. PMI was further categorized by location (anterolateral PMI and/or posteromedian PMI) and extent (partial (≤50 % hyperenhanced papillary myocardium) or complete (>50 % hyperenhanced papillary myocardium) on LGE short-axis images (Fig. 1) [11].

Left atrial volume was calculated using the summation of slices method multiplied by slice thickness. Left ventricular end-diastolic diameter (LVEDD) and left ventricular end-systolic diameter (LVESD) were measured in the short-axis view at mid-LV level. Additionally, the systolic sphericity index (SSI) (ratio of LV width to length) was measured in the four-chamber view at end-systole. Interpapillary muscle distance (IPMD) was measured in the short-axis view at end-systole. On the stack of short-axis cines, the endocardial and epicardial borders were outlined in end-systolic and end-diastolic images. Left ventricular end-diastolic volume (LVEDV) and left ventricular end-systolic volume (LVESV) were calculated using the summation of slice method multiplied by slice thickness. LVEF was calculated as LVEF = 100 % × (LVEDV-LVESV)/LVEDV. Regional LV contractile function was graded with the wall motion score index (WMSI) using a 17-segment, 5-point scoring system (1 = normal contraction; 2 = hypokinesia; 3 = akinesia; 4 = dyskinesia; 5 = aneurysmatic).

**Fig. 2:**
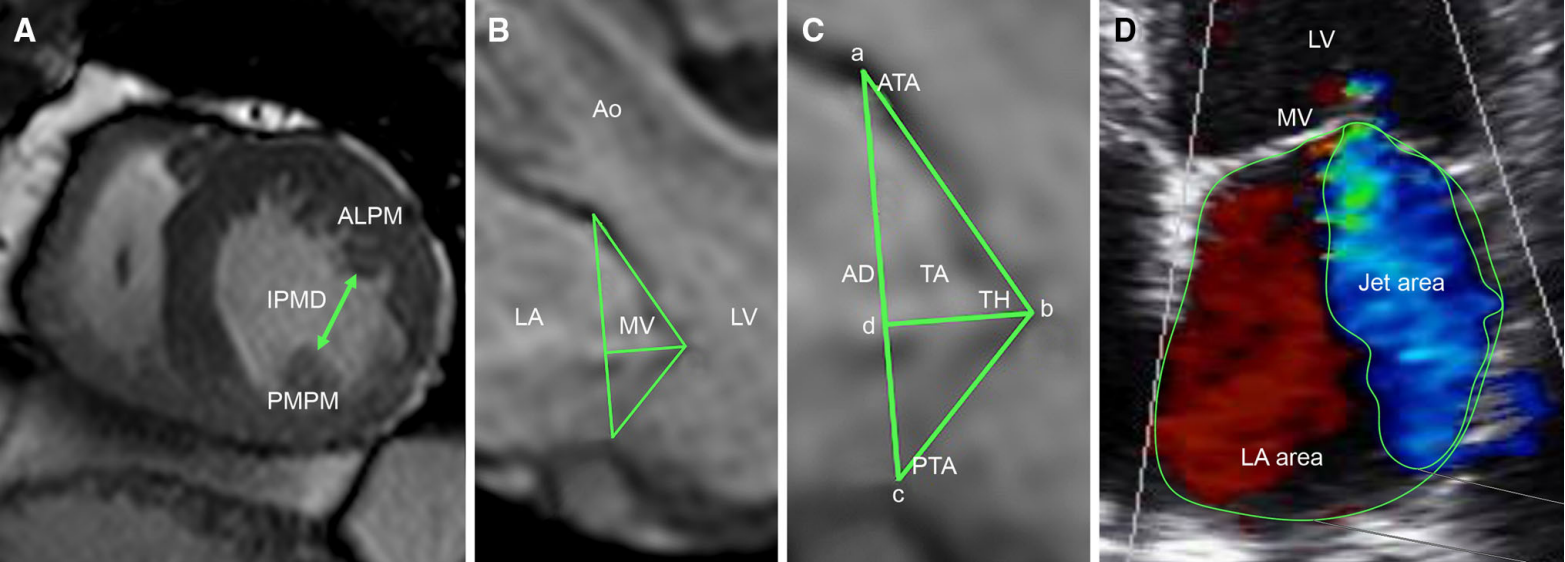
Evaluation of interpapillary muscle distance, mitral valve geometry, and CIMR severity. **A** evaluation of interpapillary muscle distance measured with cardiac MRI in the short-axis view (end-systolic). **B**, **C** Evaluation of mitral valve geometry with cardiac MRI in the 3-chamber view (mid-systolic); mitral annular diameter (*a*–*c*), tethering height (*d*–*b*), tethering area (area enclosed by *a*–*b*–*c*), posterior tethering angle (angle between *c*–*a* and *c*–*b*), anterior tethering angle (angle between *a*–*c* and *a*–*b*). **D** Evaluation of CIMR severity with TTE in the apical four-chamber view (mid-systolic) (jet area/LA area). *AD* annular diameter, *ALPM* anterolateral papillary muscle, *Ao* aorta, *ATA* anterior tethering angle, *CIMR* chronic ischemic mitral regurgitation, *IPMD* interpapillary muscle distance, *LA* left atrium, *LV* left ventricle, *MV* mitral valve, *PMPM* posteromedian papillary muscle, *PTA* posterior tethering angle, *TA* tethering area, *TH* tethering height

Mitral annular diameter, tethering height (distance between the leaflet coaptation point and the mitral annular plane), tethering area (area enclosed between the annular plane and the mitral leaflets) posterior tethering angle, and anterior tethering angle were measured in the 3-chamber view (mid-systolic) (Fig. 2) [9].

### Echocardiographic analysis

TTE was performed with commercially available equipment (Vivid-7, General Electric, Horten, Norway) with a phased array transducer. CIMR was defined as MR caused by MI in the absence of structural mitral valve abnormalities and present 4 months after PCI. Based on the echocardiography guidelines [17–19], the severity of MR was scored as no or trace (grade 1+), mild (grade 2+), moderate (grade 3+), or severe (grade 4+) as defined by jet area divided by left atrial area measured with TTE in the apical four-chamber view (Table 1). CIMR was considered present if jet area/left atrial (LA) area ≥10 % (≥grade 2+).

**Table 1:** Echocardiographic CIMR severity grading

Grade	Grade specification	Jet area/left atrial area (%)^a^
1+	No or trace	<10
2+	Mild	10–20
3+	Moderate	20–40
4+	Severe	≥40

*CIMR* chronic ischemic mitral regurgitation

^a^Color-doppler apical four-chamber view, mid-systolic

### Statistics

Continuous variables were expressed as mean ± SD. Categorical variables were expressed as percentages. Comparisons between groups were performed using Pearson’s Chi-square test or Fisher`s exact test as appropriate for categorical variables and the independent samples *t* test or Mann–Whitney *U* test as appropriate for continuous variables. Univariate variables with *P* <0.10 were included in the multivariate analysis. Age and gender were forced in all multivariate models. Multivariate logistic regression analyses by means of a forward stepwise algorithm (cut-off for entry and removal set at a significance level of 0.05) were performed to identify independent predictors of PMI and CIMR. Odds ratios were reported with 95 % confidence intervals (CI). Goodness-of-fit of the final logistic regression models was assessed with the Hosmer–Lemeshow statistic.

All calculations were performed using commercially available statistical packages (IBM SPSS Statistics 21.0; IBM Corporation, Chicago, IL, USA and Stats Direct 2.8.0; StatsDirect Ltd, Chesire, UK). Statistically significant differences were defined as *P* < 0.05.

## Results

### Study Population

**Fig. 3:**
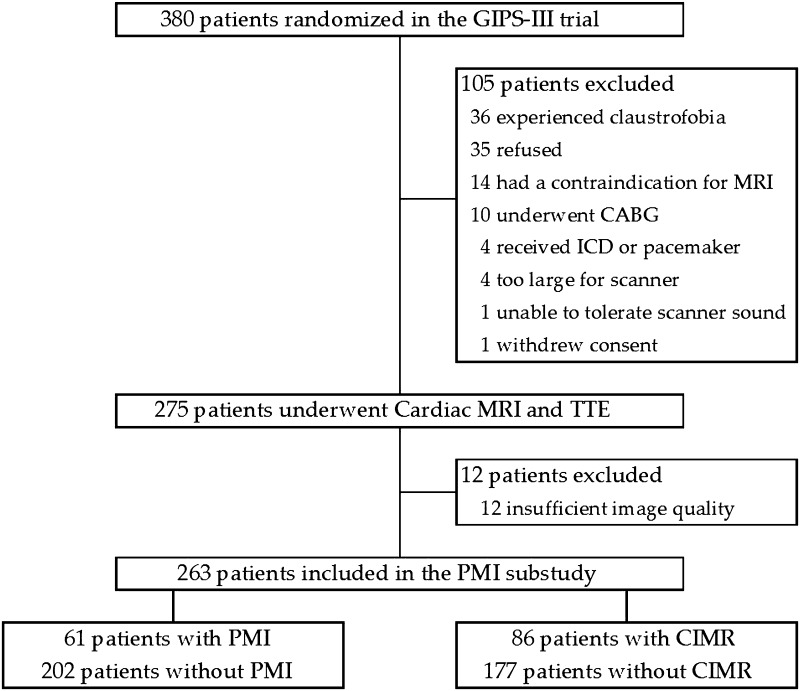
Flowchart for the PMI substudy. *CABG* coronary artery bypass grafting, *CIMR* chronic ischemic mitral regurgitation, *GIPS* glycometabolic intervention as adjunct to primary percutaneous intervention in ST elevation myocardial infarction, *ICD* implantable cardioverter defibrillator, *MRI* magnetic resonance imaging, *PMI* papillary muscle infarction, *TTE* transthoracic echocardiography

A flowchart for this substudy is shown in Fig. 3. Patient characteristics are shown in Tables 2 and 3. PMI was present in 61 patients (23 %) and CIMR was present in 86 patients (33 %). In patients with PMI, 52 % had CIMR, and in patients without PMI, 27 % had CIMR (*P* < 0.001).

**Table 2:** Patient characteristics

Variable^a^	Total (*n* = 263)	Papillary muscle infarction (PMI)			Chronic ischemic MR (CIMR)		
		PMI (*n* = 61)	No PMI (*n* = 202)	*P* value	CIMR (*n* = 86)	No CIMR (*n* = 177)	*P* value
Age, years	57.8 ± 11.5	59.3 ± 11.7	57.4 ± 11.5	0.262	63.2 ± 11.2	55.2 ± 11.7	<0.001
Female	57 (22 %)	12 (20 %)	45 (22 %)	0.665	22 (26 %)	35 (20 %)	0.284
Body mass index, kg/m^2^	27.0 ± 3.6	26.9 ± 3.2	27.0 ± 3.7	0.812	26.4 ± 3.2	27.3 ± 3.7	0.060
Cardiovascular related history							
Hypertension	76 (29 %)	17 (28 %)	59 (29 %)	0.840	26 (30 %)	50 (28 %)	0.739
Dyslipidemia	164 (62 %)	39 (64 %)	125 (62 %)	0.772	44 (51 %)	120 (56 %)	0.009
Smoking	184 (70 %)	40 (66 %)	144 (71 %)	0.394	50 (58 %)	134 (76 %)	0.004
Stroke	1 (1 %)	1 (2 %)	0 (0 %)	0.232	1 (1 %)	0 (0 %)	0.327
Peripheral artery disease	0 (0 %)	0 (0 %)	0 (0 %)	−	0 (0 %)	0 (0 %)	−
Previous PCI	4 (2 %)	0 (0 %)	4 (2 %)	0.576	0 (0 %)	4 (2 %)	0.307
Ischemic time, min	203 ± 148	211 ± 160	201 ± 145	0.649	202 ± 159	204 ± 143	0.924
Maximum CK level, U/L	2071 ± 1928	3188 ± 1892	1734 ± 1812	<0.001	2687 ± 2179	1772 ± 1721	<0.001
Number of diseased coronary arteries				0.247			0.767
One-vessel disease	190 (72 %)	39 (64 %)	151 (75 %)	−	64 (74 %)	126 (71 %)	−
Two-vessel disease	62 (24 %)	19 (31 %)	43 (21 %)	−	18 (21 %)	44 (25 %)	−
Three-vessel disease	11 (4 %)	3 (5 %)	8 (4 %)	−	4 (5 %)	7 (4 %)	−
Infarct-related artery				<0.001			0.424
Left main	0 (0 %)	0 (0 %)	0 (0 %)	−	0 (0 %)	0 (0 %)	−
Left anterior descending coronary artery	106 (40 %)	6 (10 %)	100 (50 %)	−	34 (40 %)	72 (41 %)	−
Left circumflex coronary artery	44 (17 %)	28 (46 %)	16 (8 %)	−	18 (21 %)	26 (15 %)	−
Right coronary artery	113 (43 %)	27 (44 %)	86 (43 %)	−	34 (40 %)	79 (45 %)	−
Thrombus aspiration	245 (93 %)	57 (93 %)	188 (71 %)	1.000	78 (91 %)	167 (94 %)	0.271
Stent placement	259 (98 %)	61 (100 %)	198 (98 %)	0.576	84 (98 %)	175 (99 %)	0.599
Infarct-related artery TIMI flow							
Preintervention grade				0.190			0.037
0	151 (57 %)	42 (69 %)	109 (41 %)	−	55 (64 %)	96 (54 %)	−
1	17 (6 %)	2 (3 %)	15 (7 %)	−	5 (6 %)	12 (7 %)	−
2	45 (17 %)	9 (15 %)	36 (18 %)	−	18 (21 %)	27 (15 %)	−
3	50 (19 %)	8 (13 %)	42 (21 %)	−	8 (9 %)	42 (24 %)	−
Postintervention grade				0.555			0.441
2	17 (6 %)	5 (8 %)	12 (6 %)	−	7 (8 %)	10 (6 %)	−
3	246 (94 %)	56 (92 %)	190 (94 %)	−	79 (92 %)	167 (94 %)	−
Myocardial blush grade				0.809			0.391
0	5 (2 %)	1 (2 %)	4 (2 %)	−	3 (3 %)	2 (1 %)	−
1	18 (7 %)	3 (5 %)	15 (7 %)	−	8 (9 %)	10 (6 %)	−
2	55 (21 %)	15 (25 %)	40 (20 %)	−	18 (21 %)	37 (21 %)	−
3	183 (70 %)	42 (69 %)	141 (70 %)	−	57 (66 %)	126 (71 %)	−
Randomized to metformin treatment	130 (49 %)	31 (51 %)	99 (49 %)	0.804	40 (47 %)	90 (51 %)	0.509

*CIMR* chronic ischemic mitral regurgitation, *CK* creatine phosphokinase, *PCI* percutaneous coronary intervention, *PMI* papillary

muscle infarction, *TIMI* thrombolysis in myocardial infarction

^a^Data are presented as mean ± standard deviation or number (%)

**Table 3:** Cardiac MRI and TTE Data

Variable^a^	Total (*n* = 263)	Papillary muscle infarction (PMI)			Chronic Ischemic MR (CIMR)		
		PMI (*n* = 61)	No PMI (*n* = 202)	*P* value	CIMR (*n* = 86)	No CIMR (*n* = 177)	*P* value
Time from infarct to TTE, days	124 ± 13	125 ± 12	124 ± 12	0.318	126 ± 15	123 ± 11	0.102
Time from infarct to CMR, days	125 ± 10	126 ± 9	124 ± 9	0.131	126 ± 8	124 ± 10	0.163
Infarct size, % LV hyperenhancement	9.0 ± 7.7	12.9 ± 6.8	7.7 ± 7.6	<0.001	11.6 ± 8.1	7.6 ± 7.1	<0.001
Infarct location							
Anterior	134 (51 %)	29 (48 %)	105 (52 %)	0.543	45 (52 %)	89 (50 %)	0.756
Inferior	212 (81 %)	59 (97 %)	143 (71 %)	<0.001	75 (87 %)	137 (77 %)	0.059
Lateral	132 (50 %)	48 (79 %)	84 (42 %)	<0.001	49 (57 %)	83 (47 %)	0.125
Papillary muscle infarction	61 (23 %)	61 (100 %)	−	−	32 (37 %)	29 (16 %)	<0.001
Posteromedian PMI	52 (20 %)	52 (85 %)	−	−	28 (33 %)	24 (14 %)	<0.001
Incomplete^b^	13 (5 %)	13 (21 %)	−	−	5 (6 %)	8 (5 %)	0.763
Complete^b^	39 (15 %)	39 (64 %)	−	−	23 (27 %)	16 (9 %)	<0.001
Anterolateral PMI	19 (7 %)	19 (31 %)	−	−	8 (9 %)	11 (6 %)	0.364
Incomplete^b^	7 (3 %)	7 (11 %)	−	−	3 (3 %)	4 (2 %)	0.686
Complete^b^	12 (5 %)	12 (20 %)	−	−	5 (6 %)	7 (4 %)	0.535
Combined PMI (complete/incomplete^b^)	10 (4 %)	10 (16 %)	−	−	4 (5 %)	6 (3 %)	0.733
LA and LV geometry and function							
LA volume, ml	58.8 ± 18.6	60.4 ± 18.4	58.3 ± 18.6	0.480	64.1 ± 19.6	56.2 ± 17.5	0.002
LV end-diastolic diameter, mm	49.7 ± 5.6	50.5 ± 6.0	49.4 ± 5.5	0.213	49.9 ± 5.6	49.5 ± 5.6	0.620
LV end-diastolic volume, ml	193.6 ± 44.6	206.5 ± 44.8	189.7 ± 43.9	0.010	194.0 ± 43.4	193.4 ± 45.3	0.921
LV end-systolic diameter, mm	33.2 ± 6.2	35.0 ± 7.5	32.7 ± 5.7	0.013	34.3 ± 7.0	32.7 ± 5.8	0.055
LV end-systolic volume, ml	90.2 ± 33.7	105.1 ± 40.1	85.6 ± 30.1	<0.001	93.3 ± 35.4	88.7 ± 32.8	0.294
Systolic sphericity index, %	46.7 ± 6.2	48.3 ± 6.5	46.2 ± 6.0	0.016	48.0 ± 6.7	46.0 ± 5.8	0.011
Interpapillary muscle distance, mm	12.5 ± 4.5	14.8 ± 5.3	11.8 ± 4.0	<0.001	13.9 ± 5.0	11.8 ± 4.1	<0.001
LVEF, %	54.3 ± 8.1	50.2 ± 8.9	55.6 ± 7.4	<0.001	52.8 ± 9.6	55.0 ± 7.2	0.038
Wall motion score index	1.25 ± 0.28	1.30 ± 0.29	1.23 ± 0.28	0.120	1.32 ± 0.30	1.21 ± 0.26	0.008
CIMR severity	1.6 ± 1.0	2.2 ± 1.2	1.5 ± 0.9	<0.001	3.0 ± 0.7	1.0 ± 0.0	<0.001
CIMR Grade				<0.001			−
Grade 1+ (none or trace)	177 (67 %)	29 (48 %)	148 (73 %)	−	−	177 (100 %)	−
Grade 2+ (mild)	25 (10 %)	8 (13 %)	17 (8 %)	−	25 (29 %)	−	−
Grade 3+ (moderate)	39 (15 %)	10 (16 %)	29 (14 %)	−	39 (45 %)	−	−
Grade 4+ (severe)	22 (8 %)	14 (23 %)	8 (4 %)	−	22 (26 %)	−	−
Mitral valve geometry							
Annular diameter, mm	30.6 ± 3.7	31.0 ± 3.2	30.4 ± 3.9	0.293	30.7 ± 4.0	30.5 ± 3.6	0.658
Tethering height, mm	8.2 ± 2.0	8.8 ± 1.9	8.0 ± 2.0	0.005	8.9 ± 2.1	7.8 ± 1.8	<0.001
Tethering area, mm^2^	13.1 ± 4.0	14.3 ± 3.6	12.7 ± 4.1	0.005	14.3 ± 4.4	12.5 ± 3.7	0.001
Posterior tethering angle, °	40.7 ± 9.1	43.8 ± 8.9	39.8 ± 8.9	0.002	43.2 ± 9.3	39.6 ± 8.8	0.003
Anterior tethering angle, °	21.8 ± 5.8	22.5 ± 5.5	21.5 ± 5.9	0.253	23.3 ± 6.2	21.0 ± 5.5	0.003

*(CI)MR* (chronic ischemic) mitral regurgitation, *LA* left atrium, *LV(EF)* left ventricular (ejection fraction), *MRI* magnetic resonance imaging, *PMI* papillary muscle infarction, *TTE* transthoracic echocardiography

^a^Data are presented as mean ± standard deviation or number (%)

^b^Complete PMI: >50 % hyperenhancement on short-axis images; incomplete PMI: ≤50 % hyperenhancement on short-axis images

### PMI and the infarct-related artery

PMI was found in 61 patients (23 %). Posteromedian PMI was found in 42 patients (69 %), Anterolateral PMI was found in nine patients (15 %), and combined PMI was found in ten patients (16 %). An overview of the IRA according to type of PMI is provided in Table 4.

**Table 4:** PMI type and IRA

PMI type	Infarct-related artery		
	LAD (%)	LCx	RCA
Any type of PMI	10	46	44
Posteromedian PMI	0	38	62
Anterolateral PMI	67	33	0
Combined PMI	0	90	10

*IRA* infarct-related artery, *LAD* left anterior descending coronary artery; *LCx* left circumflex coronary artery, *PMI* papillary muscle infarction, *RCA* right coronary artery

### Predictors of PMI

Univariate and multivariate logistic regression analyses of PMI are shown in Tables 2, 3, and 5. Multivariate analysis revealed infarct size, inferior MI, and circumflex infarct-related artery as independent predictors of PMI. The Hosmer–Lemeshow goodness-of-fit test was non-significant, indicating that this multivariate model is a good fit (*χ* ^2^ = 13.85, *df* = 8, *P* = 0.086).

**Table 5:** Predictors of PMI by univariate and multivariate logistic regression analysis

Variable	Univariate analysis		Multivariate analysis	
	OR (95 % CI)	*P* value	OR (95 % CI)	*P* value
Age, years	1.01 (0.99–1.04)	0.261	−	
Female	0.85 (0.42–1.74)	0.665	−	
Maximum CK level, U/L	1.00 (1.00–1.00)	<0.001	−	
Infarct-related artery LCx	9.86 (4.82–20.21)	<0.001	8.21 (3.80–17.74)	<0.001
Preintervention TIMI flow grade 0	1.89 (1.03–3.47)	0.041	−	
Infarct size, % LV hyperenhancement	1.09 (1.05–1.13)	<0.001	1.09 (1.04–1.13)	<0.001
Inferior MI	9.45 (2.23–40.09)	0.002	4.64 (1.04–20.62)	0.044
Lateral MI	5.19 (2.64–10.17)	<0.001	−	

*CI* confidence interval, *CK* creatinine phosphokinase, *LCx* circumflex coronary artery, *LV* left ventricle, *MI* myocardial infarction, *OR* odds ratio, *PMI* papillary muscle infarction, *TIMI* thrombolysis in myocardial infarction

### Predictors of CIMR

Univariate and multivariate logistic regression analyses of CIMR are shown in Tables 2, 3, and 6. Multivariate analysis revealed age, infarct size, tethering height, and interpapillary muscle distance as independent predictors of CIMR. The Hosmer–Lemeshow goodness-of-fit test was non-significant, indicating that this multivariate model is a good fit (*χ* ^2^ = 4.87, *df* = 8, *P* = 0.772).

**Table 6:** Predictors of CIMR by univariate and multivariate logistic regression analysis

Variable	Univariate analysis		Multivariate analysis	
	OR (95 % CI)	*P* value	OR (95 % CI)	*P* value
Age, years	1.07 (1.04–1.10)	<0.001	1.08 (1.04–1.11)	<0.001
Female	1.40 (0.76–2.57)	0.285	−	
Maximum CK level, U/L	1.00 (1.00–1.00)	0.001	−	
Preintervention TIMI flow grade <3	3.03 (1.36–6.79)	0.007	−	
Infarct size, % LV hyperenhancement	1.07 (1.03–1.11)	<0.001	1.09 (1.03–1.16)	0.003
Inferior MI	1.99 (0.97–4.11)	0.062	−	
PMI	3.02 (1.67–5.46)	<0.001	−	
LA volume, ml	1.02 (1.01–1.04)	0.003	−	
LV end-systolic diameter, mm	1.04 (1.00–1.09)	0.058	−	
Systolic sphericity index, %	1.06 (1.01–1.10)	0.012	−	
Interpapillary muscle distance, mm	2.79 (1.54–5.04)	0.001	3.32 (1.31–8.42)	0.011
LVEF, %	0.97 (0.94–1.00)	0.040	−	
Wall motion score index	3.60 (1.38–9.41)	0.009	−	
Tethering height, mm	19.03 (4.57–79.22)	<0.001	19.30 (3.28–113.61)	0.001
Tethering area, mm^2^	3.04 (1.56–5.93)	0.001	−	
Posterior tethering angle, °	1.05 (1.02–1.08)	0.003	−	
Anterior tethering angle, °	1.07 (1.02–1.12)	0.004	−	

*CI* confidence interval, *CIMR* chronic ischemic mitral regurgitation, *CK* creatinine phosphokinase, *LA* left atrial, *LV(EF)* left ventricular (ejection fraction), *MI* myocardial infarction, *OR* odds ratio, *PMI* papillary muscle infarction, *TIMI* thrombolysis in myocardial infarction

## Discussion

LGE cardiac MRI is the technique of choice for detecting scar tissue and fibrosis formation after MI and the high resolution of this technique permits careful delineation of partial or complete involvement of the PM in the infarcted area [3, 11, 20]. The incidence of PMI in this study was 23 %, but this number varies among different studies, ranging between 14 and 53 % [3, 11, 21–25]. The variability may be explained by differences in patient characteristics and treatment or by differences in cardiac MRI technique [22, 25, 26]. Due to improvements in reperfusion therapy more recent studies report a lower incidence of PMI [3, 11]. Most studies with LGE cardiac MRI to assess PMI have been performed early after infarction (several days to approximately 1 month) [3, 11, 23]. A distinct advantage of our study is the fact that PMI and LV assessment were performed 4 months after MI [27]. Because the majority of myocardial remodeling occurs over the course of this period, we were able to provide a more reliable assessment of the effect of PMI on myocardial remodeling and CIMR.

In patients with PMI, the posteromedian PM was involved in 85 % and the anterolateral PM was involved in 31 %. The posteromedian PM is known to be more prone to ischemia/infarction (and rupture) due to its dependence on single blood supply from the posterior descending coronary artery (which is either derived from the LCx or from the RCA) [28, 29]. The anterolateral PM is less vulnerable to ischemia/rupture due to its dual blood supply from the LAD and LCx [28, 29]. This is supported by the findings from this study (Table 4). PMI is usually limited to one PM, but both PMs may be involved in up to one-third of patients [3, 11, 21–25]. In this study, both PMs were infarcted in 16 % of patients with PMI. Thus, PM perfusion and infarction patterns are similar across different PMI studies.

Infarct size, inferior MI, and the LCx as IRA were independent predictors of PMI in this study. Two other studies also showed that in patients with PMI infarct size is generally larger on MRI, that myocardial scar most often involves the lateral and inferior walls, and that the IRA is most often the RCA or LCx [3, 11].

PMI also has been shown to have a strong (negative) prognostic value [3], which could be related to ventricular arrhythmias [30], but may also be related to accompanying LV dysfunction and development of CIMR [1, 5]. The prognostic value of PMI was not tested in this study, but provides an interesting subject for future studies.

CIMR was defined as MR 4 months after PCI for STEMI present on TTE with a jet area/LA area of ≥10 % (grade 2+) in the mid-systolic apical four-chamber view. Different studies use different time intervals to characterize IMR as chronic. The minimum interval is usually 6 weeks. We chose a 4 month period to make sure the majority of myocardial remodeling has occurred [27] and that IMR can truly be considered chronic. Other CIMR imaging techniques such as exercise echocardiography can provide additional useful information about the dynamic component of CIMR because it has the potential to unmask higher degrees of MR [31]. In addition, other validated parameters for CIMR severity assessment, such as regurgitant volume and effective regurgitant orifice area using the PISA (proximal isovelocity surface area) method might provide a more reliable assessment of CIMR severity [17, 32]. However, these imaging modalities or echocardiographic parameters were not available for patients from the GIPS-III trial. Differences in the timing of MR assessment and the technique used to assess its presence, and the parameter used to quantify its severity may explain some of the variability in the reported incidence of MR after MI.

CIMR increases the risk of heart failure and mortality in a graded fashion according to MR severity [1, 2]. Because CIMR has such a negative impact on prognosis, it is important to identify which mechanisms cause and which parameters predict CIMR. Especially, when these parameters can be therapeutically influenced or surgically corrected in moderate or severe CIMR.

The exact mechanism for the development of CIMR after MI remains a subject of debate [9]. Both annular dilatation (and flattening) (Carpentier type I dysfunction) and leaflet tethering (Carpentier type IIIb dysfunction) reduce leaflet coaptation and render the mitral valve incompetent in CIMR [9]. The relative contributions of both mechanisms may differ in patients, because several studies have shown a high degree of variability in the pathologic anatomy of CIMR with annular and leaflet distortions demonstrating a high degree of regional heterogeneity [33, 34]. This confirms the complex nature of CIMR and shows that multiple mechanisms interact to produce CIMR.

In a quest to unravel these mechanisms and their underlying pathological abnormalities, several studies have tried to shed light on the precise role of PM involvement in the development of CIMR [3, 11, 23, 24, 35, 36]. This study shows that PMI rate is significantly higher in patients with CIMR compared to patients without CIMR and that patients with PMI have significantly more severe CIMR compared to patients without PMI. However, in multivariate analysis, PMI was not an independent predictor of CIMR. From other contrast-enhanced MRI studies [3, 11, 23, 24] and several animal studies [35, 36] it also became clear that PMI is not an (independent) predictor of CIMR. Tethering height and interpapillary muscle distance appeared to be the strongest independent (geometric) predictors of CIMR. Tethering angles, tethering area and SSI were all associated with CIMR, but did not independently predict CIMR. This confirms findings from two other studies that showed that CIMR is related to outward displacement of the PMs and impairment of lateral shortening between them rather than to global LV dilatation [37, 38]. Thus, development of CIMR is mainly related to infarct size, LV remodeling with PM displacement, and mitral valve tethering, rather than to PMI itself. This finding may also have implications for the mechanism-based surgical correction of moderate or severe CIMR.

Undocumented pre-existing mitral valve disease may have been present at the time of MI in some patients although none of the patients had a history of (organic) mitral valve disease or evidence of structural mitral valve disease on cardiac MRI or TTE. Baseline post-MI MRI data were not available in this study. Other limitations are related to methods of CIMR quantification. Alternative validated methods for CIMR severity assessment, including regurgitant volume and effective regurgitant orifice area were not available in this study. Inherent limitations of two-dimensional imaging, such as viewing plane selection and regional asymmetry or localized annular distortions, may have biased results. Future studies with three-dimensional imaging may have the potential to overcome some of these limitations.

In conclusion, our findings indicate that 4 months after primary PCI for STEMI CIMR rates are higher in patients with PMI, but PMI is not an independent predictor of CIMR. The geometric parameters tethering height and interpapillary muscle distance are the strongest independent predictors of CIMR. Inferior infarction and infarction in the circumflex coronary artery are independent predictors of PMI.

## References

[1] Grigioni F, Enriquez-Sarano M, Zehr KJ, Bailey KR, Tajik AJ (2001). Ischemic mitral regurgitation: long-term outcome and prognostic implications with quantitative Doppler assessment. Circulation.

[2] Grigioni F, Detaint D, Avierinos JF, Scott C, Tajik J, Enriquez-Sarano M (2005). Contribution of ischemic mitral regurgitation to congestive heart failure after myocardial infarction. J Am Coll Cardiol.

[3] Eitel I, Gehmlich D, Amer O (2013). Prognostic relevance of papillary muscle infarction in reperfused infarction as visualized by cardiovascular magnetic resonance. Circ Cardiovasc Imaging.

[4] Godley RW, Wann S, Rogers EW, Feigenbaum H, Weyman AE (1981). Incomplete mitral leaflet closure in patients with papillary muscle dysfunction. Circulation.

[5] Bursi F, Enriquez-Sarano M, Nkomo VT (2005). Heart failure and death after myocardial infarction in the community: the emerging role of mitral regurgitation. Circulation.

[6] Gueret P, Khalife K, Jobic Y (2008). Echocardiographic assessment of the incidence of mechanical complications during the early phase of myocardial infarction in the reperfusion era: a French multicentre prospective registry. Arch Cardiovasc Dis.

[7] Hickey MS, Smith LR, Muhlbaier LH (1988). Current prognosis of ischemic mitral regurgitation. Implications for future management. Circulation.

[8] Frantz E, Weininger F, Oswald H, Fleck E, Vetter HO, Hetzer R, Schmutzler H (1991). Predictors for mitral regurgitation in coronary artery disease. Ischemic mitral incompetence.

[9] Bouma W, van der Horst IC, Wijdh-den Hamer IJ (2010). Chronic ischaemic mitral regurgitation. Current treatment results and new mechanism-based surgical approaches. Eur J Cardiothorac Surg.

[10] Horstkotte JC, Horstkotte M, Beucher H, Felderhoff T, Boekstegers P (2015). Percutaneous mitral valve repair as rescue procedure after post myocardial infarction papillary muscle rupture and acute cardiogenic shock. Clin Res Cardiol.

[11] Chinitz JS, Chen D, Goyal P (2013). Mitral apparatus assessment by delayed enhancement CMR: relative impact of infarct distribution on mitral regurgitation. JACC Cardiovasc Imaging.

[12] Lexis CP, van der Horst IC, Lipsic E (2012). Metformin in non-diabetic patients presenting with ST elevation myocardial infarction: rationale and design of the glycometabolic intervention as adjunct to primary percutaneous intervention in ST elevation myocardial infarction (GIPS)-III trial. Cardiovasc Drug Ther.

[13] Lexis CP, van der Horst IC, Lipsic E (2014). Effect of metformin on left ventricular function after acute myocardial infarction in patients without diabetes: the GIPS-III randomized clinical trial. JAMA.

[14] Haver VG, Hartman MH, Mateo Leach I (2015). Leukocyte telomere length and left ventricular function after acute ST-elevation myocardial infarction: data from the glycometabolic intervention as adjunct to primary coronary intervention in ST elevation myocardial infarction (GIPS-III) trial. Clin Res Cardiol.

[15] The TIMI IIIB Investigators (1994). Effects of tissue plasminogen activator and a comparison of early invasive and conservative strategies in unstable angina and non-Q-wave myocardial infarction. Results of the TIMI IIIB trial. Circulation.

[16] van ‘t Hof AW, Liem A, Suryapranata H, Hoorntje JC, de Boer MJ, Zijlstra F (1998). Angiographic assessment of myocardial reperfusion in patients treated with primary angioplasty for acute myocardial infarction: myocardial blush grade. Zwolle Myocardial Infarction Study Group. Circulation.

[17] Zoghbi WA, Enriquez-Sarano M, Foster E (2003). American Society of Echocardiography. Recommendations for evaluation of the severity of native valvular regurgitation with two-dimensional and Doppler echocardiography. J Am Soc Echocardiogr.

[18] Enriquez-Sarano M, Akins CW, Vahanian A (2009). Mitral regurgitation. Lancet.

[19] Nishimura RA, Otto CM, Bonow RO (2014). American College of Cardiology, American Heart Association Task Force on Practice Guidelines. 2014 AHA/ACC guideline for the management of patients with valvular heart disease: executive summary: a report of the American College of Cardiology/American Heart Association Task Force on Practice Guidelines. J Am Coll Cardiol.

[20] Bax JJ, Delgado V (2013). Papillary muscle infarction, mitral regurgitation, and long-term prognosis. Circ Cardiovasc Imaging.

[21] Hombach V, Grebe O, Merkle N (2005). Sequelae of acute myocardial infarction regarding cardiac structure and function and their prognostic significance as assessed by magnetic resonance imaging. Eur Heart J.

[22] Peters DC, Appelbaum EA, Nezafat R (2009). Left ventricular infarct size, peri-infarct zone, and papillary scar measurements: a comparison of high-resolution 3D and conventional 2D late gadolinium enhancement cardiac MR. J Magn Reson Imaging.

[23] Tanimoto T, Imanishi T, Kitabata H (2010). Prevalence and clinical significance of papillary muscle infarction detected by late gadolinium-enhanced magnetic resonance imaging in patients with ST-segment elevation myocardial infarction. Circulation.

[24] Okayama S, Uemura S, Soeda T (2011). Clinical significance of papillary muscle late enhancement detected via cardiac magnetic resonance imaging in patients with single old myocardial infarction. Int J Cardiol.

[25] Aldrovandi A, De Ridder SP, Strohm O, Cocker M, Sandonato R, Friedrich MG (2013). Detection of papillary muscle infarction by late gadolinium enhancement: incremental value of short-inversion time vs. standard imaging. Eur Heart J Cardiovasc Imaging.

[26] Yang Y, Connelly K, Graham JJ (2011). Papillary muscle involvement in myocardial infarction: initial results using multicontrast late-enhancement MRI. J Magn Reson Imaging.

[27] Pokorney SD, Rodriguez JF, Ortiz JT, Lee DC, Bonow RO, Wu E (2012). Infarct healing is a dynamic process following acute myocardial infarction. J Cardiovasc Magn Reson.

[28] Estes EH, Dalton FM, Entman ML, Dixon HB, Hackel DB (1966). The anatomy and blood supply of the papillary muscles of the left ventricle. Am Heart J.

[29] Voci P, Bilotta F, Caretta Q, Mercanti C, Marino B (1995). Papillary muscle perfusion pattern. A hypothesis for ischemic papillary muscle dysfunction. Circulation.

[30] Bogun F, Desjardins B, Crawford T (2008). Post-infarction ventricular arrhythmias originating in papillary muscles. J Am Coll Cardiol.

[31] Lancellotti P, Lebrun F, Piérard LA (2003). Determinants of exercise-induced changes in mitral regurgitation in patients with coronary artery disease and left ventricular dysfunction. J Am Coll Cardiol.

[32] Grayburn PA (2008). How to measure severity of mitral regurgitation: valvular heart disease. Heart.

[33] Ryan LP, Jackson BM, Parish LM (2007). Regional and global patterns of annular remodeling in ischemic mitral regurgitation. Ann Thorac Surg.

[34] Vergnat M, Jassar AS, Jackson BM (2011). Ischemic mitral regurgitation: a quantitative three-dimensional echocardiographic analysis. Ann Thorac Surg.

[35] Mittal AK, Langston M, Cohn KE, Selzer A, Kerth WJ (1971). Combined papillary muscle and left ventricular wall dysfunction as a cause of mitral regurgitation: an experimental study. Circulation.

[36] Miller GE, Kerth WJ, Gerbode F (1968). Experimental papillary muscle infarction. J Thorac Cardiovasc Surg.

[37] Otsuji Y, Levine RA, Takeuchi M, Sakata R, Tei C (2008). Mechanism of ischemic mitral regurgitation. J Cardiol.

[38] Kalra K, Wang Q, McIver BV (2014). Temporal changes in interpapillary muscle dynamics as an active indicator of mitral valve and left ventricular interaction in ischemic mitral regurgitation. J Am Coll Cardiol.

